# Interplay between Livestock Grazing and Aridity on the Ecological and Nutritional Value of Forage in Semi-arid Mediterranean Rangelands (NE Spain)

**DOI:** 10.1007/s00267-024-01939-9

**Published:** 2024-02-01

**Authors:** Antonio I. Arroyo, Yolanda Pueyo, Olivia Barrantes, Concepción L. Alados

**Affiliations:** 1 Instituto Pirenaico de Ecología (IPE), CSIC, Av. Montañana 1005, 50059 Zaragoza, Spain; 2 Departamento de Ciencias Agrarias y del Medio Natural, Facultad de Veterinaria (Universidad de Zaragoza), C/ Miguel Servet 177, 50013 Zaragoza, Spain; 3 Instituto Agroalimentario de Aragón -IA2- (CITA-Universidad de Zaragoza), C/ Miguel Servet 177, 50013 Zaragoza, Spain

**Keywords:** Annual plant production, Grazing intensity, Middle Ebro Valley, Plant diversity, Plant C:N ratio, Plant fiber composition

## Abstract

Rangeland-based livestock production constitutes a primary source of livelihood for many inhabitants of dryland regions. Their subsistence relies heavily on maintaining the productivity, biodiversity and services of these ecosystems. Harsh environmental conditions (e.g., drought) combined with land use intensification (e.g., overgrazing) make dryland ecosystems vulnerable and prone to degradation. However, the interplay between livestock grazing intensity and aridity conditions in driving the conservation and nutritional value of forage in arid and semi-arid rangelands is still not fully understood. In this study, we performed structural equation models (SEM) to assess the simultaneous direct and indirect effects of livestock grazing intensity and aridity level on community structure, diversity, biomass, forage production, forage C:N ratio and forage fiber composition in two semi-arid Mediterranean rangelands, NE Spain. Not surprisingly, we found that higher livestock grazing intensity led to lower community plant cover, especially when combined with higher aridity. However, both increasing grazing intensity and aridity were associated with higher forage production after one year of grazing exclusion. We did not find any adverse effect of livestock grazing on plant diversity, although plant species composition differed among grazing intensity levels. On the other hand, we found an aridity-driven trade-off in regard of the nutritional value of forage. Specifically, higher aridity was associated with a decrease in the least digestible fiber fraction (i.e., lignin) and an increase in forage C:N ratio. More interestingly, we found that livestock grazing modulated this trade-off by improving the overall forage nutritional value. Altogether, our results provide further insights into the management of semi-arid Mediterranean rangelands, pointing out that maintaining traditional rangeland-based livestock production may be a sustainable option as long as rangeland conservation (e.g., community plant cover) is not severely compromised.

## Introduction

Drylands, regions where the ratio between mean annual precipitation and potential evapotranspiration (i.e., aridity index) is less than 0.65, are one of the most widely distributed biomes across the globe (Prăvălie 2016) and are inhabited by more than a third of the world’s population (Safriel et al. 2005; Reynolds et al. 2007). Rangeland-based livestock production, together with crop farming, is the most widespread land use in these regions and constitutes the basic livelihood for a considerable proportion of their inhabitants (Asner et al. 2004; Campos et al. 2018). As such, their subsistence relies heavily on maintaining the productivity, biodiversity and services (e.g., forage provisioning) of dryland ecosystems. However, increasingly harsh environmental conditions (e.g., drought) combined with drastic changes in land use (e.g., overgrazing or land abandonment) make these ecosystems vulnerable and prone to degradation (Reynolds et al. 2007; Huang et al. 2020; Burrell et al. 2020).

Excessive grazing pressure by domestic livestock negatively affects the structure and functioning of arid and semi-arid rangelands (Maestre et al. 2016; Vandandorj et al. 2017; Gaitán et al. 2018). For instance, plant community composition can be severely altered by diminishing or removing the most palatable species from the community, while favoring those that are grazing-tolerant or avoidant (Alados et al. 2003; Cipriotti et al. 2019; Oñatibia et al. 2020). Furthermore, high grazing pressure usually causes a substantial diversity loss in these ecosystems (Alados et al. 2003; Eldridge and Delgado-Baquerizo 2017; Pelliza et al. 2021), which ultimately leads to a lessening of their functioning (Maestre et al. 2016; Zhang et al. 2021). Moreover, livestock grazing can considerably reduce plant cover and biomass (Alados et al. 2003; Eldridge et al. 2016; Oñatibia et al. 2020). As vegetation cover disappears, the soil loses its protection and degradation processes such as soil erosion are enhanced (D’Odorico et al. 2019). On the other hand, land abandonment can also have negative consequences for the productivity and functioning of Mediterranean rangeland ecosystems. For example, grazing abandonment can lead to a decrease in soil fertility or promote shrub encroachment, with the associated reduction in vegetation productivity and forage provision (Peco et al. 2017). Therefore, sustainable land management practices in arid and semi-arid rangelands require the consideration of appropriate livestock grazing pressure in order to mitigate degradation processes, productivity and diversity loss (Zhang et al. 2021).

Aridity is also an important factor that negatively affects the structure, dynamics and functioning of dryland ecosystems (e.g., plant productivity, species diversity, soil fertility, microbial communities, etc.; Maestre et al. 2016; Berdugo et al. 2020). Future climate change scenarios foresee an overall increase in aridity on a global scale, particularly in dryland ecosystems, caused by changes in precipitation patterns and rising temperatures (IPCC 2014). In line with the predicted increase in aridity, reduced and less predictable herbaceous biomass production is projected across global rangelands (Godde et al. 2020). Consequently, the economic viability of rangeland-based livestock production in arid and semi-arid Mediterranean rangelands is seriously threatened (Nardone et al. 2010; Godde et al. 2020), as forage abundance is essential to reduce the intake of supplementary feed and its associated costs (Herrero et al. 2013). This situation would force the gradual abandonment of traditional rangeland-based livestock production or the overexploitation of less favored grazing areas, thus, promoting the risk of land degradation. Furthermore, although rarely assessed, climate change drivers may interact with grazing pressure with potentially devastating consequences for warmer drylands (Maestre et al. 2022). Accordingly, aridity has been reported to exacerbate grazing-induced rangeland degradation (Oñatibia et al. 2020). Therefore, it is crucial to conduct more studies assessing the combined effects of livestock grazing intensity and aridity level on the conservation and forage production of arid and semi-arid rangelands to ensure proper management of their resources (Maestre et al. 2022).

Beyond forage abundance, forage nutritional value (i.e., forage chemical and fiber composition) is also affected by livestock grazing. Livestock can directly enhance forage nutritional value by promoting the growth of younger plant shoots, which have higher leaf:stem ratio, lower C:N ratio, richer non-cell wall proteins and soluble carbohydrates, etc. (Zhai et al. 2018; García-Baquero et al. 2021). In addition, grazing by livestock may indirectly influence forage nutritional value by inducing changes in plant species composition. In this respect, grazing can promote annuals over perennial plants by creating gaps in the plant cover that favor seed germination of annual species (Noy-Meir et al. 1989; Díaz et al. 2007), although higher grazing pressure can also benefit woody species in some arid and semi-arid rangelands (Eldridge et al. 2013). On the other hand, aridity can also impact the nutritional forage value of arid and semi-arid Mediterranean rangelands. For instance, high temperatures tend to increase lignification of plant tissues, thereby decreasing forage digestibility (Van Soest 1994). Furthermore, aridity is a key factor affecting plant community composition by enabling those species with traits related to drought resistance to persist and dominate in the community (e.g., higher tissue lignification, smaller leaf size, lower N and higher C:N ratio, etc.; Oñatibia et al. 2020), leading to lower palatability. However, the interplay between livestock grazing intensity and aridity level in determining the forage nutritional value of arid and semi-arid rangelands has seldom been addressed in the bibliography.

In this study, we aimed to assess the concurrent direct and indirect effects of livestock grazing intensity and aridity level on the ecological value (in terms of community structure, diversity, forage production and biomass) and nutritional forage value (in terms of forage C:N ratio and fiber composition) of two semi-arid Mediterranean rangelands in NE Spain. Overall, we expected to find additive effects of grazing intensity and aridity level with respect to forage availability and forage production, but opposite effects with respect to forage nutritional value. In particular, we hypothesized that (i) grazing by domestic livestock would directly affect forage availability, production and nutritional value. More specifically, we expected that higher grazing intensity would lead to a reduction in plant cover, thus, rising vulnerability to degradation and threatening community conservation, while enhancing forage production and forage nutritional value. In addition, we hypothesized that (ii) grazing would impact community diversity and composition and, indirectly, vegetation biomass, forage production and forage nutritional value. On the other hand, we hypothesized that (iii) aridity level would directly affect forage availability, production and nutritional value. We expected that Mediterranean rangelands in more arid environments would exhibit lower plant cover than those in less arid environments. We also expected that an increase in aridity would cause a reduction in forage nutritional value, due to a higher plant C:N ratio and lower fiber digestibility. Finally, we also hypothesized that (iv) aridity level would alter community structure and composition which, in turn, would mediate changes in forage availability, production and nutritional value.

## Material and Methods

### Study Area

This study was conducted in the Middle Ebro Valley (NE Spain; Supplementary Fig. S1). This area, characterized by a semi-arid Mediterranean climate, is one of the most arid regions in Spain. Mean annual precipitation ranges between 300 and 500 mm, from the center to the north and south of the basin, with summer being the driest season. The average annual temperature in the region is around 15 °C with a pronounced continentality. Mean maximum summer temperatures easily exceed 30 °C, while average minimum winter temperatures are around 0 °C (data obtained from the Digital Climatic Atlas of Aragón; https://www.aragon.es/-/atlas-climatico-de-aragon; Cuadrat et al. 2007). Silty soils and gypsum outcrops predominate in the study area. The topography is characterized by flat-bottomed valleys surrounded by low hills, with elevation ranging between approximately 200 and 700 m.a.s.l. The main human activity in the region involves traditional agro-pastoral land use, with rainfed winter cereal crops and extensive sheep production (Rasa aragonesa; <0.07 LU ha^−1^ year^−1^; Pueyo 2005).

Two locations were selected within the study area, Mediana de Aragón (41° 25’ 38” N, 0° 44’ 53” W) and Leciñena (41° 48’ 29” N, 0° 34’ 7” W; Supplementary Fig. S1). These two locations had similar lithology and edaphic conditions (i.e., gypsiferous soils with low organic matter content and basic pH), topography and grazing regime, but they differed in the aridity level. Mediana de Aragón is among the most arid locations in the study area (mean annual precipitation 364 ± 19 mm year^−1^ and mean annual temperature 14.5 ± 0.3 °C; Table 1), while Leciñena presents less arid conditions (mean annual precipitation 467 ± 3.9 mm year^−1^ and mean annual temperature 13.7 ± 0.1 °C; Table 1). Vegetation in Mediana de Aragón consisted of relatively open scrubland dominated by dwarf shrubs (e.g., *Gypsophila struthium* subsp. *hispanica* (Willk.) G.López, *Thymus vulgaris* L. or *Herniaria fruticosa* L.), perennial grasses (e.g., *Brachypodium retusum* (Pers.) Beauv., *Koeleria vallesiana* (Honckeny) Gaud. or *Stipa lagascae* Roem. et Schultes) and several ephemeral herbs. In Leciñena, the plant community consisted of a relatively dense shrub-steppe dominated by shrubs (e.g., *Cistus clusii* Dunal, *Genista scorpius* (L.) DC. in Lam. et DC., *Gypsophila struthium* subsp. *hispanica* (Willk.) G.López, *Ononis tridentata* L., *Rosmarinus officinalis* L. or *Thymus vulgaris* L.), accompanied by some grasses and annuals. In both locations, vegetation appeared clumped in patches within a matrix of bare soil.

**Table 1:** Location, grazing intensity, climatic (i.e., mean annual precipitation, mean annual temperature and aridity index for the period 1971–2020) and biotic (i.e., species richness and the relative abundance of shrubs and dwarf shrubs, perennial grasses and annuals) parameters for each study plot.

Study plot	Location	Grazing intensity	Mean annual precipitation (mm)	Mean annual temperature (°C)	Aridity index^a^ (P/PET)	Species richness	Plant type abundance (%)		
							Shrubs and dwarf shrubs	Perennial grasses	Annuals
M1L	Mediana	Low	364.6	14.4	0.30	18	89.22	5.66	5.12
M1M	Mediana	Medium	366.1	14.3	0.32	27	51.71	35.96	12.33
M1H	Mediana	High	365.6	14.3	0.31	15	74.23	23.93	1.84
M2L	Mediana	Low	399.1	13.9	0.35	35	44.66	34.08	21.26
M2M	Mediana	Medium	377.1	14.5	0.30	28	66.03	23.42	10.56
M2H	Mediana	High	375.3	14.5	0.31	26	42.56	27.18	30.26
M3L	Mediana	Low	338.9	14.9	0.28	22	71.32	25.18	3.49
M3M	Mediana	Medium	346.0	14.8	0.28	22	47.83	35.04	17.14
M3H	Mediana	High	344.8	14.8	0.29	20	67.47	23.49	9.04
L1L	Leciñena	Low	466.2	13.6	0.42	20	96.71	2.27	1.02
L1M	Leciñena	Medium	466.1	13.7	0.40	14	88.66	11.18	0.16
L1H	Leciñena	High	463.8	13.7	0.38	16	92.90	4.30	2.80
L2L	Leciñena	Low	471.2	13.7	0.45	12	92.01	7.99	0.00
L2M	Leciñena	Medium	465.6	13.9	0.38	13	98.76	0.41	0.83
L2H	Leciñena	High	463.7	13.9	0.39	20	82.41	14.96	2.62
L3L	Leciñena	Low	461.8	13.7	0.42	17	94.93	3.28	1.79
L3M	Leciñena	Medium	471.8	13.6	0.43	21	63.84	32.32	3.84
L3H	Leciñena	High	472.3	13.6	0.41	30	72.58	14.62	12.79

^a^Lower values indicate higher aridity

Climatic data was obtained from the Digital Climatic Atlas of Aragón (https://www.aragon.es/-/atlas-climatico-de-aragon; Cuadrat et al. 2007)

### Experimental Design and Field Surveys

Three livestock shelters were selected at each location. The shelters are mainly used by the animals to stay overnight, while they graze extensively in the surrounding area daily, and have been harboring flocks of sheep (Rasa aragonesa) all year round for decades. Sheep flocks in Mediana de Aragón and Leciñena were around 900 and 1200 animals per shelter, respectively. Three sites with different grazing intensities, low (L), medium (M) and high (H), were identified around each shelter (i.e., 2 locations × 3 shelters × 3 grazing intensities = 18 sites; Supplementary Fig. S1). Grazing intensities were defined after jointly considering interviews with shepherds, distance to the shelter and the amount of sheep droppings (Supplementary Fig. S2A). A 40 × 40 m square study plot was demarcated at each site. Eight 1 × 1 m sampling quadrats were then established within each study plot (i.e., four at the vertices, a fifth one in the center, and the remaining three placed among the former; Supplementary Fig. S2B).

Vegetation in the study plots was surveyed in the spring of 2017. Specifically, the canopy cover (%) of every plant species found within five sampling quadrats (i.e., the four in the corners and the one in the center; denoted as P in Supplementary Fig. S2B) was recorded. In order to facilitate visual estimation of plant species cover, the sampling quadrat area was divided into 100 squares (10 cm^2^) by using strings. In addition, plant species were classified into woody (i.e., *chamaephytes* and *nanophanerophytes*) and non-woody (i.e., perennial grasses, annuals, *geophytes* and *hemicryptophytes*) species. Moreover, forage biomass samples (i.e., those parts that would potentially be consumed by livestock) were harvested, plucking them out by hand, from every plant individual found within the three remaining sampling quadrats (denoted as *P* in Supplementary Fig. S2B). Samples from the same quadrat were mixed together in order to obtain a representative sample. Finally, the central sampling quadrat was fenced off to prevent further grazing. Next, forage biomass was harvested by hand within the fenced quadrat in spring 2018 and spring 2019 to provide a measure of annual forage production.

### Plant Community Characteristics and Forage Nutritional Value

For each study plot, plant diversity was computed as the effective number of species from the Shannon-Wiener index (Jost 2006). This can be interpreted as the number of species of equal abundance required to produce a given value in the traditional Shannon-Wiener index, which is easily interpretable (e.g., a plant community with an effective species number of 10 is twice as diverse as another community with an effective number of species of 5; Jost 2006). Calculation of diversity used plant cover to approximate species abundance. Furthermore, community structure was summarized, for each sampling quadrat, as follows:

$${{{\mathrm{Woody}}}}\,{{{\mathrm{index}}}} = \frac{{\left( {Woody\,species\,cover - Non\,woody\,species\,cover} \right)}}{{Woody + Non\,woody\,species\,cover}}$$

This index ranges between 1 and −1. Positive values indicate a dominance of woody species, where 1 is the exclusive presence of woody species in the quadrat. Negative values indicate a dominance of non-woody species, where −1 is the absence of woody species in the sampling quadrat. Values around 0 indicate a similar predominance of woody and non-woody species.

In arid and semi-arid plant communities, visual estimation of plant cover is a suitable indicator of aboveground plant biomass (Flombaum and Sala 2007, 2009; Ónodi et al. 2017). Accordingly, aboveground plant biomass was estimated for each sampling quadrat as the sum of the cover of every plant species occurring within it. On the other hand, forage production was measured as the forage dry biomass produced in the fenced off sampling quadrat after one year of grazing exclusion. Because different sites had different plant cover, annual forage production was normalized to the total plant cover of the central sampling quadrat.

Forage biomass samples were taken to the laboratory on the same day as they were harvested and oven-dried at 60 °C for 48 h. Dry biomass samples (i.e., those collected outside and/or prior to grazing enclosures) were then ground to a powder and stored in zip-lock bags at −18 °C until chemical analyses were performed. Nutritional forage value was determined, on the one hand, by measuring the total C and N content of the samples using a LECO CN elemental analyzer to compute the plant C:N ratio and, on the other hand, by analyzing the fiber composition of the samples following the procedure proposed by Van Soest et al. (1991). In this analysis, samples were subjected to sequential chemical digestions to split the cell wall components into several fractions (i.e., ash-free neutral detergent fiber, NDF, acid detergent fiber, ADF, and acid detergent lignin, ADL). Cellulose (i.e., ADF − ADL), hemicellulose (i.e., NDF − ADF) and lignin (i.e., ADL) were estimated from the different fiber components. A fiber index was then computed as follows:

$${{{\mathrm{Fiber}}}}\,{{{\mathrm{index}}}} = \frac{{\left( {\left( {cellulose + hemicellulose} \right)} \right. - \left. {lignin} \right)}}{{\left( {cellulose + hemicellulose} \right. + \left. {lignin} \right)}}$$

This index ranges between 1 and −1. Positive values indicate that cell walls were composed mainly of cellulose and hemicellulose (i.e., the most digestible fiber fraction), while negative values indicate that lignin (i.e., the indigestible fraction) was the major cell wall component, thus, lowering overall fiber digestibility. Values around 0 indicate a similar proportion between lignin and more digestible fiber components.

### Statistical Analyses

Given that different variables were measured in different sampling quadrats, it was first necessary to obtain data at the study plot level for every variable, so they could be compared. Thus, while plant diversity and forage production were computed directly at the plot level, mean study plot values were obtained for the remaining variables (i.e., woody index, aboveground biomass, plant C:N ratio and fiber index; Supplementary Table S1).

To assess the hypothesized direct and indirect causal relationships between livestock grazing and aridity level and community structure, diversity, aboveground biomass, forage production and forage nutritional value, we constructed structural equation models (SEM) using the *piecewieSEM* package (Lefcheck 2016) in R 4.0.3 software (R Core Team 2021). Unlike traditional SEM analysis, the piecewise SEM approach allows for the inclusion of non-independence structures among samples and can accommodate smaller data sets (Shipley 2009). In particular, we generated four initial SEM models with the hypothesized causal relationships among variables (Supplementary Fig. S3) and fitted linear mixed-effect models (LMMs) for each component using the *nlme* package (Pinheiro et al. 2020). Livestock shelter was set as a random intercept effect in the models. Among the fixed effects, categorical grazing intensity was coded as 1 (Low), 2 (Medium) and 3 (High), while aridity level (i.e., location) was coded as 1 (Leciñena; less arid) and 2 (Mediana; more arid), thus yielding a single coefficient in the SEM models and simplifying the interpretation of the effect as the expected change produced by moving from one category to another. Annual forage production and the C:N ratio were log-transformed to meet model assumptions. We checked the correlation among all measured variables in order to find potential collinearity (Supplementary Fig. S4).

We used tests of directional separation (Shipley 2009) and chi-squared tests of Fisher’s C statistic (Shipley 2013) to assess the overall fit of the SEM models and to determine whether non-hypothesized missing paths should be included or current paths should be removed, conditional on the existing causal relationship specified (Shipley 2013; Lefcheck 2016). Accordingly, a new causal relationship between community structure and diversity was specified (Supplementary Fig. S3), while the weakest path (i.e., with the largest *p* value) was removed sequentially until all remaining paths were significant (*p* < 0.1). When multiple models were possible, the model with the lowest AICc (i.e., an AICc difference ≥ 2) was selected as the best overall model (Shipley 2013). This process reduces SEM models’ complexity by including the most important relationships and removing most of the non-significant paths (Grace et al. 2015). Finally, we obtained the standardized size effects, the significance of each relationship and the variation explained for every response variable (Lefcheck 2016).

To test whether plant species composition changes across grazing intensities and environmental conditions, we performed non-metric multidimensional scaling (NMDS) and permutational multivariate analysis of variance (999 permutations) using Bray-Curtis dissimilarity, with the *metaMDS* and *adonis* functions, respectively, of the *vegan* package (Oksanen et al. 2020) in R 4.0.3 software (R Core Team 2021). Significant differences in species composition among specific pairs of grazing intensities were tested by performing pairwise comparisons using the *pairwise.adonis2* function. In addition, vectors of measured variables (i.e., woody index, diversity, plant cover, annual forage production, plant C:N ratio and fiber index) were fitted with the *envfit* function to represent the direction of maximum change in such variables. Livestock shelter was specified as a blocking variable using the *strata* argument as a control for its potential influence on species composition.

## Results

**Fig. 1:**
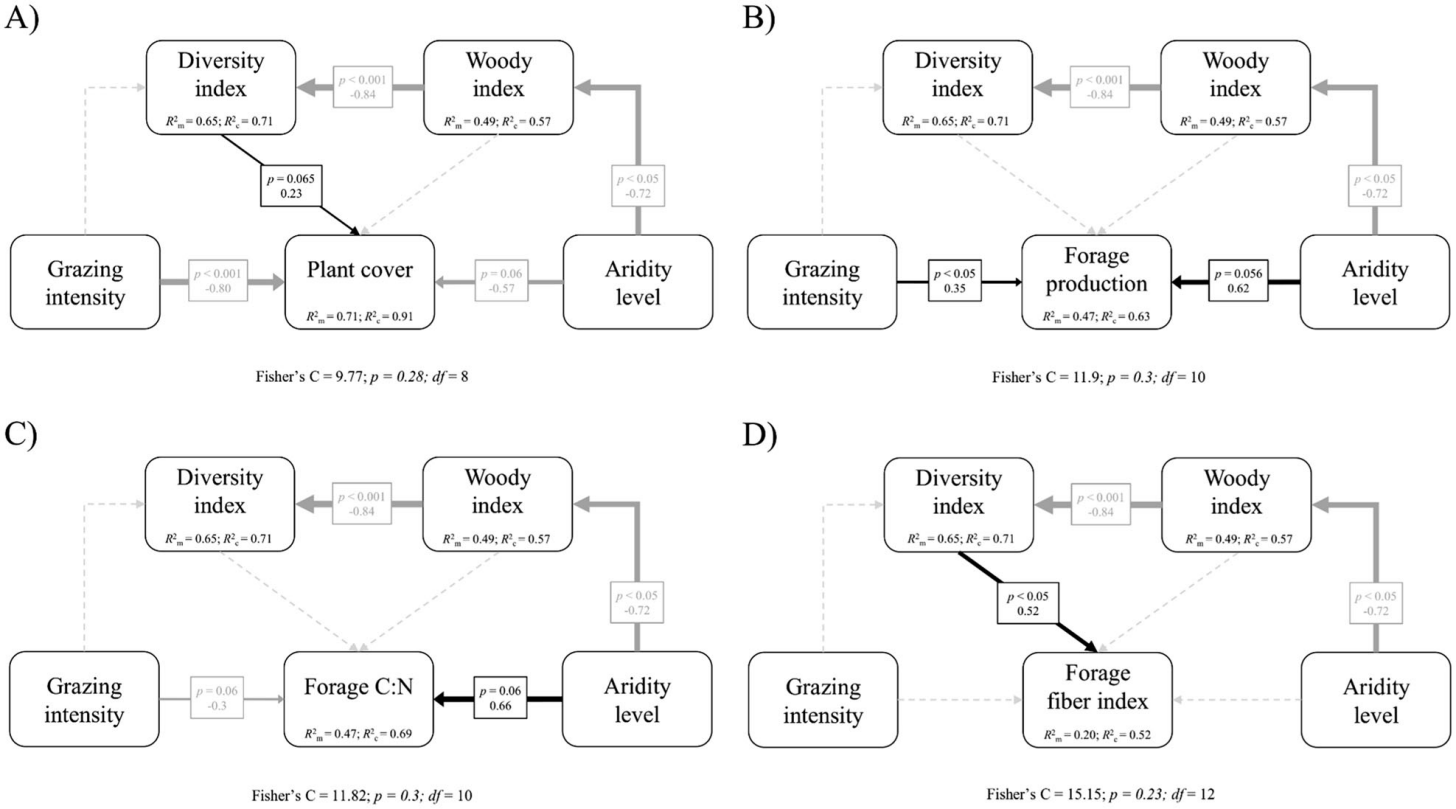
Path diagrams showing the effects of livestock grazing intensity and environmental conditions on plant community structure, diversity, (**A**) biomass, (**B**) annual forage production and nutritional value (i.e., (**C**) forage C:N ratio and (**D**) fiber composition) as derived from the best-fit piecewise SEM models. Livestock grazing intensity and aridity level were modeled as ordinal variables. Black solid arrows indicate positive causal relationships. Gray solid arrows indicate negative causal relationships. Dotted lines indicate non-significant (*p* > 0.1) hypothesized causal relationships. Standardized regression coefficients and their significance level are given over each arrow. Marginal R^2^ (based on the variance of the fixed effects) and conditional R^2^ (based on the variance of both the fixed and random effects) are given within the response variable boxes. Arrows widths are proportional to path coefficients

Selected SEM models fitted the data satisfactorily and explained a high proportion of the variance of the response variables (Fig. 1A–D). Overall, our analysis revealed additive effects of livestock grazing and environmental conditions on the ecological value of the studied semi-arid Mediterranean rangelands. We found that livestock grazing directly affected plant cover and forage production. Specifically, increasing livestock grazing intensity was associated with lower plant cover (Fig. 1A), while annual forage production increased with higher grazing intensity (Fig. 1B). Similarly, we found a direct negative marginal relationship between aridity level and plant cover (Fig. 1A), while forage production marginally increased with increasing aridity (Fig. 1B). The direct positive effect of aridity level on annual forage production was greater than that of grazing intensity, while the opposite pattern was found for plant cover (Fig. 1A, B). Furthermore, the negative effect of aridity level on plant cover was partially compensated by a net positive indirect effect. Specifically, higher aridity was associated with a lower dominance of woody species, which in turn increased plant diversity, and thus, plant cover (Fig. 1A). No indirect effects between grazing intensity and plant cover nor forage production were retained in the selected SEM models (Figs. 1A, B; Supplementary Fig. S5).

On the other hand, selected SEM models showed mainly contrasting effects of livestock grazing and environmental conditions on the nutritional forage value of our semi-arid Mediterranean rangelands. We found a direct positive marginal relationship between aridity level and plant C:N ratio (Fig. 1C) and a net positive indirect effect of aridity level on fiber index through changes in community structure and diversity (Fig. 1D). The effect of aridity level worsening plant C:N ratio was greater than its effect enhancing fiber composition (Fig. 1C, D). Interestingly, this effect was partially compensated by a direct marginal effect of livestock grazing on plant C:N ratio. More specifically, plant C:N ratio decreased as livestock grazing intensity increased (Fig. 1C). No indirect effects, via changes in plant diversity, between livestock grazing intensity and the variables related to the nutritional forage value were retained in the selected SEM models (Figs. 1C, D; Supplementary Fig. S5).

**Fig. 2:**
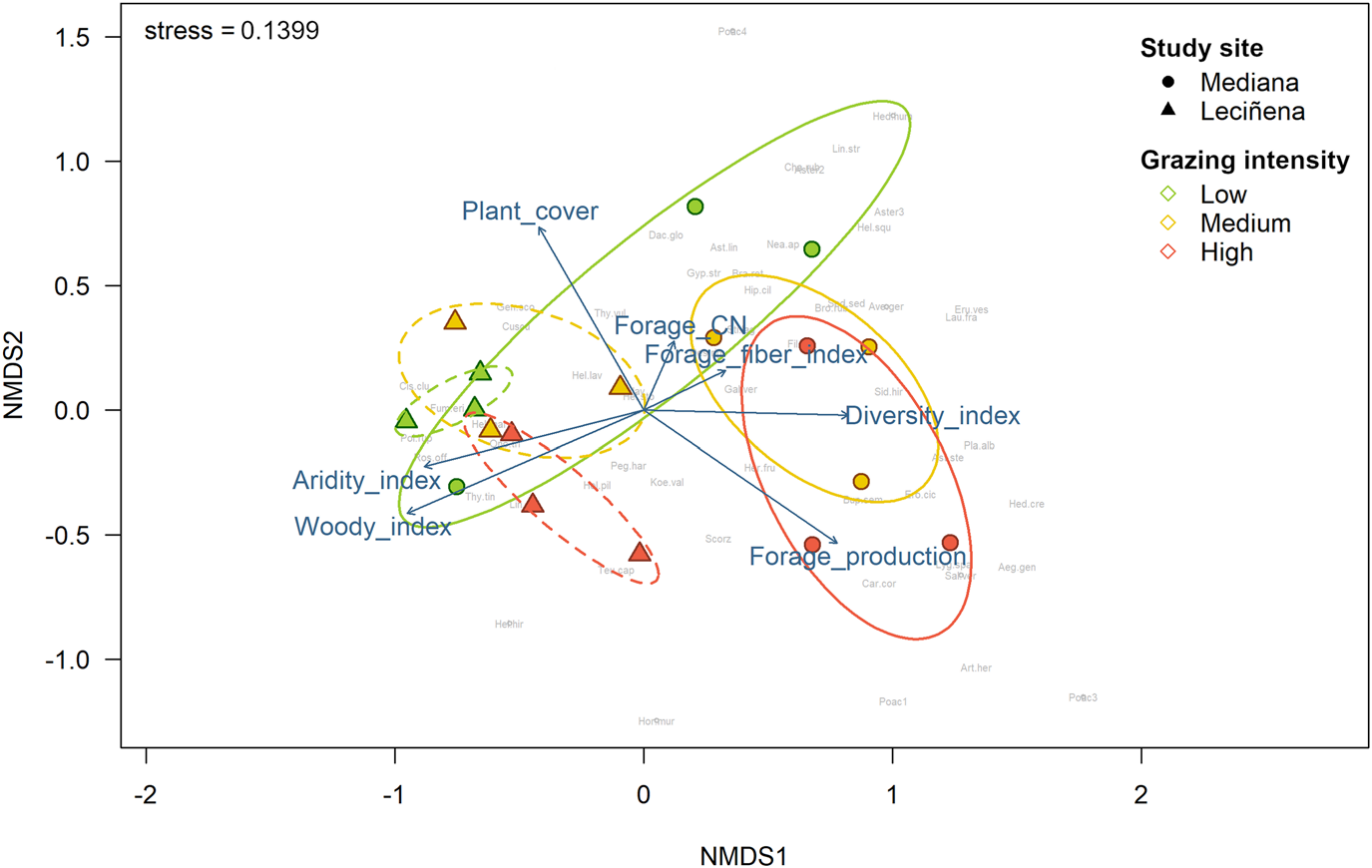
Non-metric multidimensional scaling (NMDS) ordination of study plots´ plant species composition using Bray-Curtis dissimilarity. Colors and symbols highlight different grazing intensities and sites, respectively. Ellipses indicate 95% confidence intervals (dashed and solid lines used for Leciñena and Mediana, respectively). Arrows show the correlation between measured variables and ordination axis scores. Note that lower values of the aridity index actually indicate higher aridity conditions. Full Latin names for the plant species labels are given in Supplementary Table S2

Plant species composition changed significantly among livestock grazing intensities (F_2,12_ = 1.51, *p* < 0.01; Fig. 2; Supplementary Table S2). We found a significant difference in plant species composition between low and high grazing intensities, while the medium intensity showed an intermediate composition. Accordingly, the 95% confidence ellipses of low and high grazing intensities did not overlap in the NMDS plot (Fig. 2). Not surprisingly, the direction of maximum change in aboveground plant biomass approximately matched the direction of segregation of study plots by livestock grazing intensity, with larger aboveground biomass values correlating with lower grazing intensities (Fig. 2).

Plant species composition also changed significantly between environmental conditions (F_1,12_ = 6.13, *p* < 0.05; Fig. 2; Supplementary Table S2). In accordance, the NMDS plot showed that species composition was noticeably different between the Mediana and Leciñena study plots, although a low grazed study plot in Mediana was represented within the Leciñena plot positions (Fig. 2). Also, the direction of the maximum change in the aridity index was approximately aligned with the segregation of study plots by location, with increasing aridity index values (which, in fact, indicate lower aridity conditions) correlating with Leciñena study plots. Finally, the interaction between grazing intensity and environmental conditions was not significant (F_2,12_ = 0.79, *p* = 0.65), indicating that differences in plant species composition among livestock grazing intensities responded similarly in both locations (Fig. 2).

## Discussion

In this study, we investigated the simultaneous direct and indirect effects of livestock grazing intensity and aridity level on community structure, diversity, biomass, forage production, C:N ratio and fiber composition in two semi-arid Mediterranean rangelands in NE Spain. As expected, we found that increasing livestock grazing intensity directly caused a reduction in community plant cover (i.e., aboveground biomass), in line with results reported in other semi-arid rangelands (Oñatibia and Aguiar 2019; Nakano et al. 2020; Pelliza et al. 2021), probably through several mechanisms such as defoliation and damage caused by trampling. Plant cover constitutes an essential biotic parameter that drives the conservation and functioning of semi-arid ecosystems (Maestre et al. 2016). For instance, plant cover plays a relevant role in regulating water runoff and sediment yield through rainfall interception and soil protection (Urgeghe et al. 2021). Also, a decrease in plant cover would necessarily lead to the loss of soil organic C and N, reducing soil fertility and productivity (An et al. 2019). Therefore, it has been argued that plant cover is an effective tool for monitoring the impact of livestock grazing on rangeland vegetation and, thus, on its vulnerability to degradation (Papanastasis et al. 2003). Vegetation-patch structure (e.g., patch size distribution) is also closely linked to degradation risk in semi-arid ecosystems (Kéfi et al. 2007; Maestre et al. 2016; Urgeghe et al. 2021). Although patch structure was not specifically considered in our study, previous research has shown that livestock grazing alters vegetation patches by increasing patch fragmentation, and by simplifying and removing vegetation patches (Saiz and Alados 2012, 2014; Oñatibia et al. 2018; Pelliza et al. 2021). Not surprisingly, we also found that higher aridity was associated with lower community plant cover, highlighting the urgent need to establish adequate management practices in these ecosystems in order to mitigate the impacts of uneven livestock grazing pressure, especially under the projected increase in aridity conditions.

Biodiversity conservation is also critical for maintaining the functioning of semi-arid ecosystems (Maestre et al. 2012; Zhang et al. 2021). In this respect, grazing disturbance is a key factor affecting rangeland plant diversity across global drylands (Olff and Ritchie 1998; Eldridge et al. 2016; Kouba et al. 2021). Specifically, in more productive environments, diversity peaks at low to intermediate grazing intensities, whereas in less productive environments, diversity typically shows a negative response to grazing intensity (Zhang et al. 2021). In our study, however, we did not find evidence of any direct effect of livestock grazing on plant diversity (i.e., as quantified by our diversity index). Unlike plant cover, it is likely that traditional livestock stocking rates in the study area were not enough to cause noticeable changes in this variable. In a recent meta-analysis, Herrero-Jáuregui and Oesterheld (2018) found that the effect of grazing on species richness was generally smaller than the effect on species composition. In accordance, we observed that increasing livestock grazing intensity changed plant species composition at both study locations. Given that grazing did not drastically modify community plant diversity (e.g., through the extinction of most palatable species), shifts in species composition could simply be due to changes in species dominances rather than species turnover. For instance, livestock grazing can reduce the size of preferred plant species, while that of less palatable species increases (Oñatibia and Aguiar 2019). Alternatively, the lack of any significant direct effect of livestock grazing on plant diversity could also be due to the long evolutionary history of grazing (Milchunas et al. 1988). In this sense, we found that after one year of grazing exclusion, forage production was enhanced in the formerly more intensively grazed plots, which highlights the resilience capacity of these rangelands to grazing. In short, our results support the idea that plant cover is a more sensitive indicator than plant diversity for tracking the conservation-degradation state in semi-arid Mediterranean rangelands, and point out that the maintenance of traditional rangeland-based livestock production could be compatible with rangeland conservation as long as community plant diversity is not hampered (Zhang et al. 2021).

As hypothesized, we observed that livestock grazing affected forage nutritional value in our semi-arid Mediterranean rangelands. Specifically, increasing grazing intensity directly improved forage nutritional value by reducing plant C:N ratio. It is well known that grazing holds plant species at an early maturity stage (e.g., by promoting younger plant shoots), when tissues are characterized by a lower fiber fraction and higher protein content (George et al. 2001; Bai et al. 2012; Zhai et al. 2018). In addition, a lower forage C:N ratio could be a result of higher N inputs from livestock. This might be the case in our study, as we found that increasing grazing intensity was associated with higher annual forage production under conditions of temporary grazing exclusion. On the other hand, increasing livestock grazing intensity could also reduce the nutritional value of forage due to selective grazing that favors less palatable material with higher fiber concentrations (Baranova et al. 2019). Nevertheless, in our study, we did not find evidence of direct or indirect effects of livestock grazing on forage fiber composition, which is consistent with the observed lack of strong livestock effects on community plant diversity. Thus, our results highlight the importance of sustaining low grazing intensities in order to maintain forage nutritional value at levels sufficient to meet the needs of livestock grazers (Baranova et al. 2019).

Furthermore, aridity conditions also determined forage nutritional value in these semi-arid Mediterranean rangelands. On the one hand, we found that higher aridity conditions led to an increase in forage fiber index (i.e., lower lignin proportion). More specifically, this effect was mediated by changes in plant community structure and composition. In fact, as in our semi-arid Mediterranean rangelands, extreme environmental conditions can favor the prevalence of annual plant species (i.e., therophytization; Ward 2009), which are characterized by a lower tissue lignification. On the other hand, forage C:N ratio was higher in those plant communities under more arid conditions (Bai et al. 2012). This result could be explained by the higher abundance of low-quality annual species and perennial grasses in these study sites. For instance, grasses usually present a large amount of stems and fibrous tissues (Amiri and Shariff 2012). Additionally, a higher plant C:N ratio in more arid environments could also be a consequence of a poorer soil nutrient pool and fertility (Plaza et al. 2018; Berdugo et al. 2020). Therefore, our findings emphasize that the effect of livestock grazing on enhancing nutritional forage value might be particularly relevant under more arid conditions, counterbalancing to some extent the reduction in forage abundance and contributing to the economic sustainability of extensive livestock grazing production systems.

The expected increase in aridity in semi-arid Mediterranean ecosystems does not put forward favorable prospects for traditional rangeland-based livestock production. Forage availability would be reduced under more aridity conditions (Giridhar and Samireddypalle 2015; Godde et al. 2020). This can have devastating consequences for the economic sustainability of an already marginal activity, as livestock feeding would require more external inputs (Bernués et al. 2011; Giridhar and Samireddypalle 2015). In addition, a lower amount of forage would imply less grazing time, which would worsen animal welfare (Rutter 2010). Therefore, an intensification of the widespread grazing abandonment in these areas could be anticipated (Bernués et al. 2011; Caballero 2015). In this respect, our results showed that reduced grazing pressure would effectively contribute to enhancing semi-arid Mediterranean rangelands conservation by promoting an increase in plant cover and biomass. Not surprisingly, other studies have found similar results (Wang et al. 2018; Miguel et al. 2020; Kouba et al. 2021). However, grazing abandonment can also have negative effects. In this line, our results suggest that grazing abandonment would cause changes in plant species composition and a reduction in forage nutritional value. Therefore, the maintenance of light-intensive grazing regimes (and their associated benefits) should be integrated into management strategies to preserve the functioning of semi-arid Mediterranean ecosystems (Peco et al. 2017; Zhang et al. 2021). Nevertheless, complementary measures, such as a return to shepherd guided grazing instead of free ranging, or the establishment of water sources to attract livestock to graze less used areas, might be necessary to cope with the upcoming increase in aridity (Bailey 2004).

## Conclusions

In conclusion, our study found that increased livestock grazing intensity led to a reduction in community plant cover, thus enhancing the vulnerability of semi-arid Mediterranean rangelands to degradation, especially in more arid environments. However, we did not find any adverse effect of livestock grazing on community plant diversity, although higher grazing intensity by domestic livestock caused changes in plant species composition. Finally, we found an aridity-driven trade-off between forage C:N ratio and forage fiber index and, more interestingly, that livestock grazing modulated it by improving the overall nutritional value of the forage. Altogether, our results provide further insights into the management of semi-arid Mediterranean rangelands, pointing out that maintaining traditional rangeland-based livestock production can be a suitable option. Nevertheless, further research is still needed to define precise sustainable livestock stocking rates and grazing intensities, as well as to assess the generality of our findings in other rangelands worldwide along an aridity gradient and over a multiyear study period.

### Supplementary information

Supplementary Information

## Data Availability

Data will be made available upon reasonable request.
