# Supplementary material for: Interplay between Livestock Grazing and Aridity on the Ecological and Nutritional Value of Forage in Semi-arid Mediterranean Rangelands (NE Spain)
Source: Environ Manage. 2024 Feb 1;73(5):1005–15. doi: 10.1007/s00267-024-01939-9 (PMC11024040; doi:10.1007/s00267-024-01939-9)
Supplement: Supplementary file 1 — Supplementary Information [file 267_2024_1939_MOESM1_ESM.docx]

**Interplay between livestock grazing and aridity on the ecological and nutritional value of forage in semi-arid Mediterranean rangelands (NE Spain)**

Environmental Management

Antonio I. Arroyo ^a,*^, Yolanda Pueyo ^a^, Olivia Barrantes ^b,c^, Concepción L. Alados ^a^

^a^ Instituto Pirenaico de Ecología (IPE), CSIC, Av. Montañana 1005, 50059, Zaragoza, Spain

^b^ Departamento de Ciencias Agrarias y del Medio Natural, Facultad de Veterinaria (Universidad de Zaragoza), C/ Miguel Servet 177, 50013, Zaragoza, Spain

^c^ Instituto Agroalimentario de Aragón -IA2- (CITA-Universidad de Zaragoza), C/ Miguel Servet 177, 50013, Zaragoza, Spain

^*^ Corresponding author. E-mail: [aiarroyo@ipe.csic.es](mailto:aiarroyo@ipe.csic.es) Telephone: +34 976 369 393 ext. 880038. Fax: +34 974 363 222

**Supplementary Information**

**Fig. S1** Location of study plots in the Middle Ebro Valley (Spain). See Table 1 for further details.

**Fig. S2** A) Number of sheep droppings (mean ± SE) per m^2^ for each grazing intensity and location. Different letters indicate significant differences among grazing intensities (*post-hoc* Tukey HSD test; *p* < 0.05). Significance of differences was tested by fitting a generalized linear mixed model (GLMM) in which grazing intensity and location were set as fixed factors, and study plot was set as a random intercept effect. A negative binomial distribution of errors was specified in the model to deal with overdispersion. B) Schematic representation of study plots and sampling quadrats.

**Table S1** Mean (± SE) for the variables of interest at the plot level. See Materials and Methods section for further details.

**Fig. S3** Initial path diagram with the hypothesized causal relationships among variables. Grazing intensity and aridity level are exogenous variables, while woody index, diversity, plant cover, production, plant C:N ratio and fiber index are endogenous variables. A total of four path diagrams were generated, one for each final variable (*i.e.*, plant cover, forage production, forage C:N ratio and forage fiber index). Directions of the arrows indicate the causal relationships between variables. Dashed arrow indicates a non-hypothesized missing path added during the evaluation of the overall models fit. See Materials and Methods section for further details.

**Fig. S4** Spearman correlation coefficients among all variables included in the SEM analysis. Blue and red colors indicate positive and negative correlations respectively, with more intense colors denoting higher correlation values. Visual representation of the correlation matrix was created with the *GGally* package in R 4.0.3.

**Fig. S5** Direct (dark colors) and indirect (light colors) effects of grazing intensity (solid bars) and aridity (dashed bars) on community plant cover, forage production, C:N ratio and fiber index, community structure and diversity at A) *p* < 0.05 and B) *p* < 0.1. Red bars indicate negative effects. Blue bars indicate positive effects. See Materials and Methods section for further details.

**Table S2** List of plant species found at the study plot level, their labels, forage value (FV) and plant cover (%).

Fig. S1


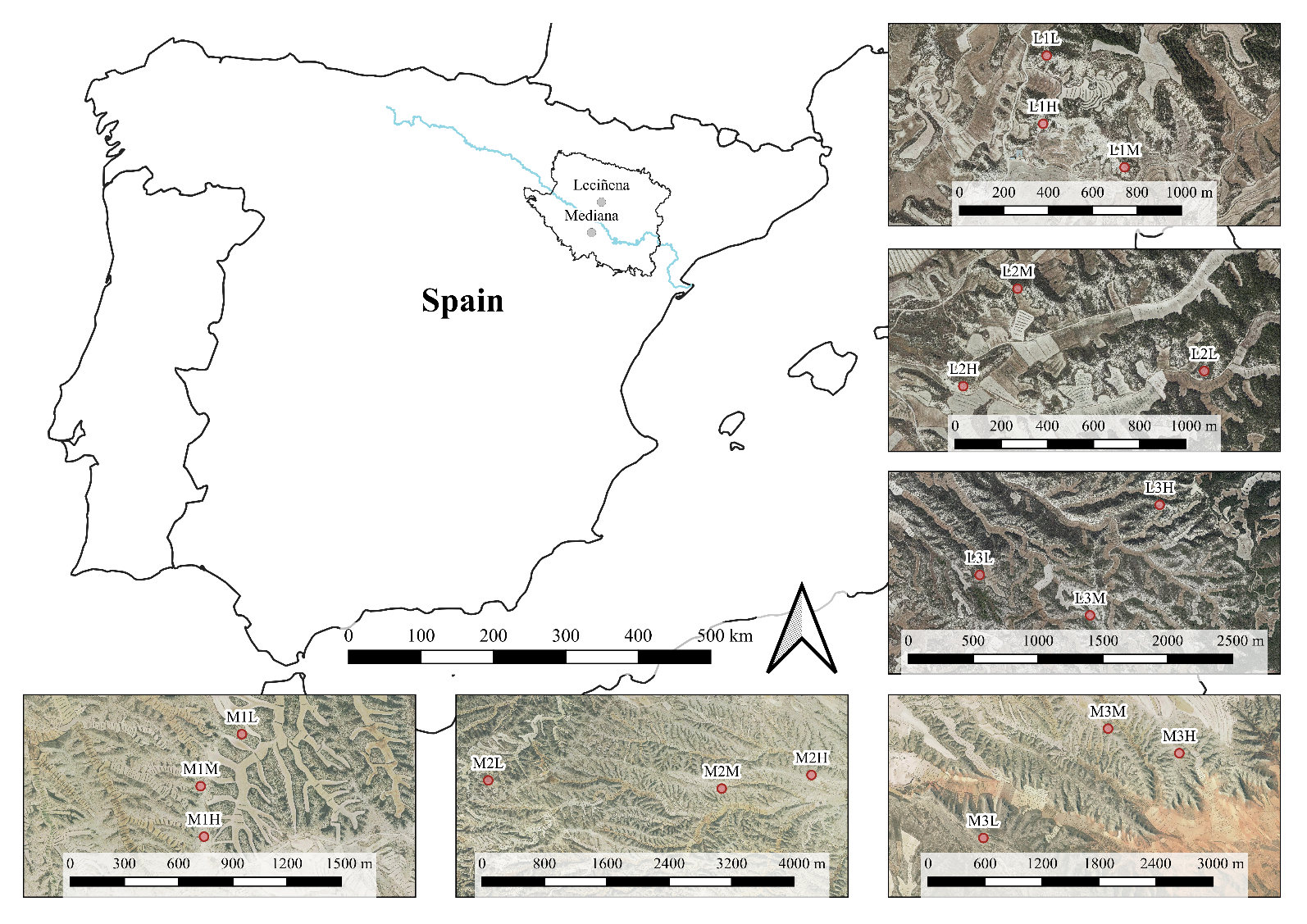


Fig. S2


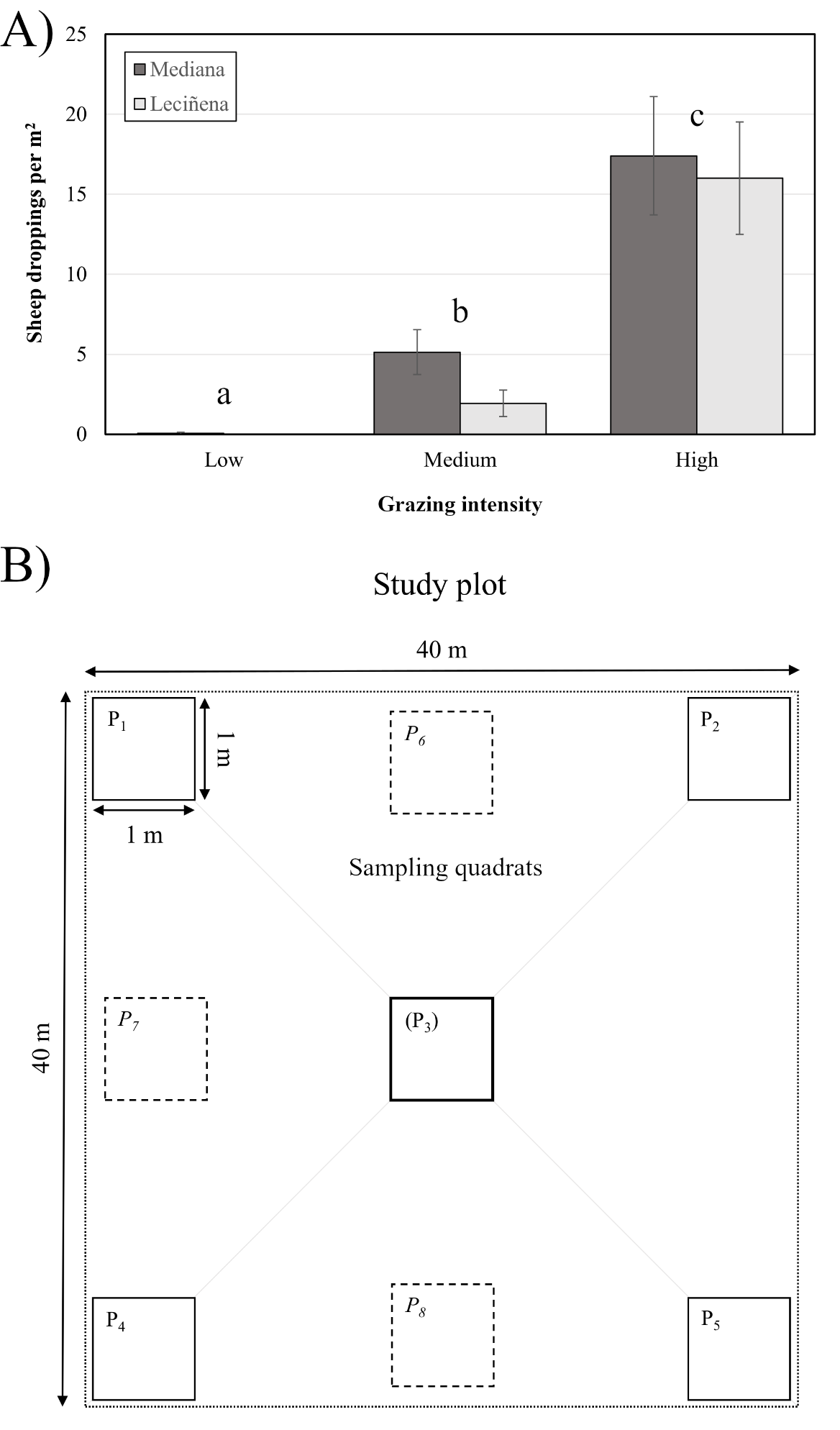


Table S1

| **Study plot** | **Location** | **Grazing intensity** | **Aridity level** | **Woody index** | **Diversity** | **Plant cover (%)** | **Forage production**  **(dry g m^-1^ year^-1^)^a^** | **Forage C:N**  **ratio** | **Forage fiber**  **index** |
| --- | --- | --- | --- | --- | --- | --- | --- | --- | --- |
| M1L | Mediana | Low | Arid/Semi-arid | 0.809 ± 0.060 | 4.039 | 37.1 ± 6.4 | 2.32 | 56.29 ± 10.41 | -0.120 ± 0.056 |
| M1M | Mediana | Medium | Arid/Semi-arid | 0.055 ± 0.129 | 16.976 | 29.4 ± 2.5 | 3.76 | 33.90 ± 2.83 | 0.381 ± 0.062 |
| M1H | Mediana | High | Arid/Semi-arid | 0.292 ± 0.238 | 11.895 | 17.1 ± 5.2 | 2.36 | 25.82 ± 3.11 | 0.380 ± 0.112 |
| M2L | Mediana | Low | Arid/Semi-arid | -0.090 ± 0.204 | 14.102 | 88.9 ± 21.0 | 1.39 | 39.63 ± 4.45 | 0.432 ± 0.120 |
| M2M | Mediana | Medium | Arid/Semi-arid | 0.214 ± 0.108 | 13.065 | 52.3 ± 14.7 | 2.90 | 34.82 ± 0.34 | 0.530 ± 0.067 |
| M2H | Mediana | High | Arid/Semi-arid | -0.170 ± 0.139 | 12.266 | 19.7 ± 5.5 | 3.44 | 30.03 ± 2.31 | 0.370 ± 0.086 |
| M3L | Mediana | Low | Arid/Semi-arid | 0.392 ± 0.276 | 7.750 | 54.4 ± 16.1 | 1.71 | 34.83 ± 3.31 | 0.461 ± 0.039 |
| M3M | Mediana | Medium | Arid/Semi-arid | -0.092 ± 0.296 | 11.602 | 39.3 ± 8.0 | 3.03 | 43.45 ± 3.00 | 0.360 ± 0.034 |
| M3H | Mediana | High | Arid/Semi-arid | 0.235 ± 0.175 | 10.377 | 17 ± 3.8 | 7.88 | 26.54 ± 2.12 | 0.687 ± 0.104 |
| L1L | Leciñena | Low | Semi-arid | 0.917 ± 0.026 | 8.239 | 88.8 ± 12.8 | 1.36 | 30.68 ± 7.49 | 0.564 ± 0.131 |
| L1M | Leciñena | Medium | Semi-arid | 0.675 ± 0.146 | 5.949 | 62.6 ± 19.8 | 0.45 | 39.18 ± 3.49 | 0.441 ± 0.046 |
| L1H | Leciñena | High | Semi-arid | 0.868 ± 0.061 | 5.218 | 46.5 ± 10.3 | 1.46 | 22.15 ± 5.59 | 0.602 ± 0.093 |
| L2L | Leciñena | Low | Semi-arid | 0.849 ± 0.082 | 4.504 | 86.4 ± 5.5 | 0.23 | 21.65 ± 1.69 | 0.281 ± 0.036 |
| L2M | Leciñena | Medium | Semi-arid | 0.981 ± 0.011 | 6.031 | 72.4 ± 12.3 | 0.76 | 21.70 ± 2.71 | 0.417 ± 0.066 |
| L2H | Leciñena | High | Semi-arid | 0.679 ± 0.111 | 6.809 | 38.3 ± 5.0 | 1.26 | 25.10 ± 6.51 | 0.422 ± 0.053 |
| L3L | Leciñena | Low | Semi-arid | 0.909 ± 0.058 | 6.296 | 67.1 ± 6.1 | 2.07 | 17.22 ± 0.82 | 0.234 ± 0.050 |
| L3M | Leciñena | Medium | Semi-arid | 0.246 ± 0.089 | 12.894 | 49.5 ± 7.0 | 2.45 | 14.99 ± 0.86 | 0.745 ± 0.032 |
| L3H | Leciñena | High | Semi-arid | 0.423 ± 0.134 | 15.622 | 38.4 ± 12.4 | 1.67 | 17.02 ± 0.71 | 0.384 ± 0.071 |

^a^ as normalized by the total plant cover of the central sampling quadrat.

Fig. S3


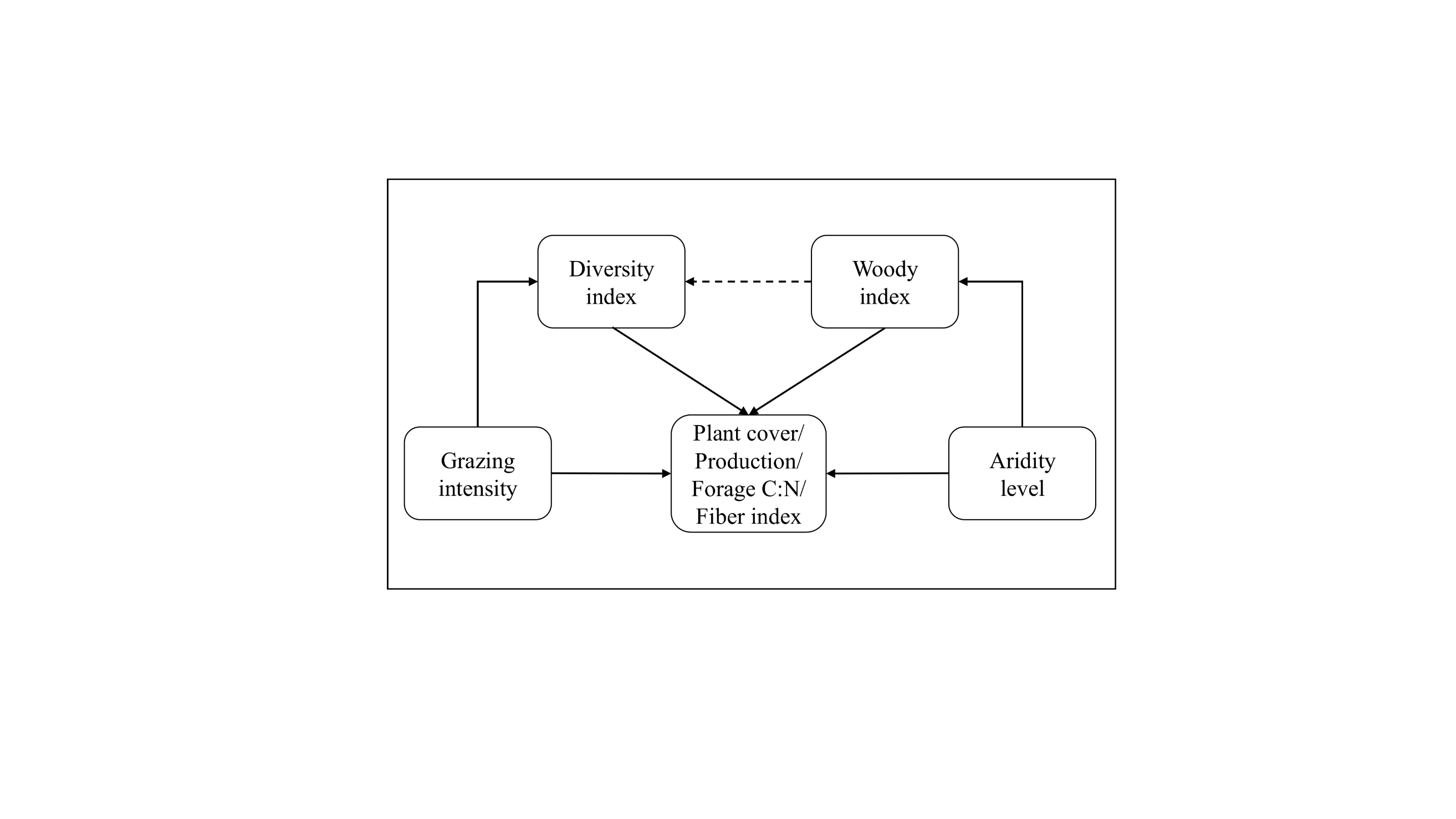


Fig. S4


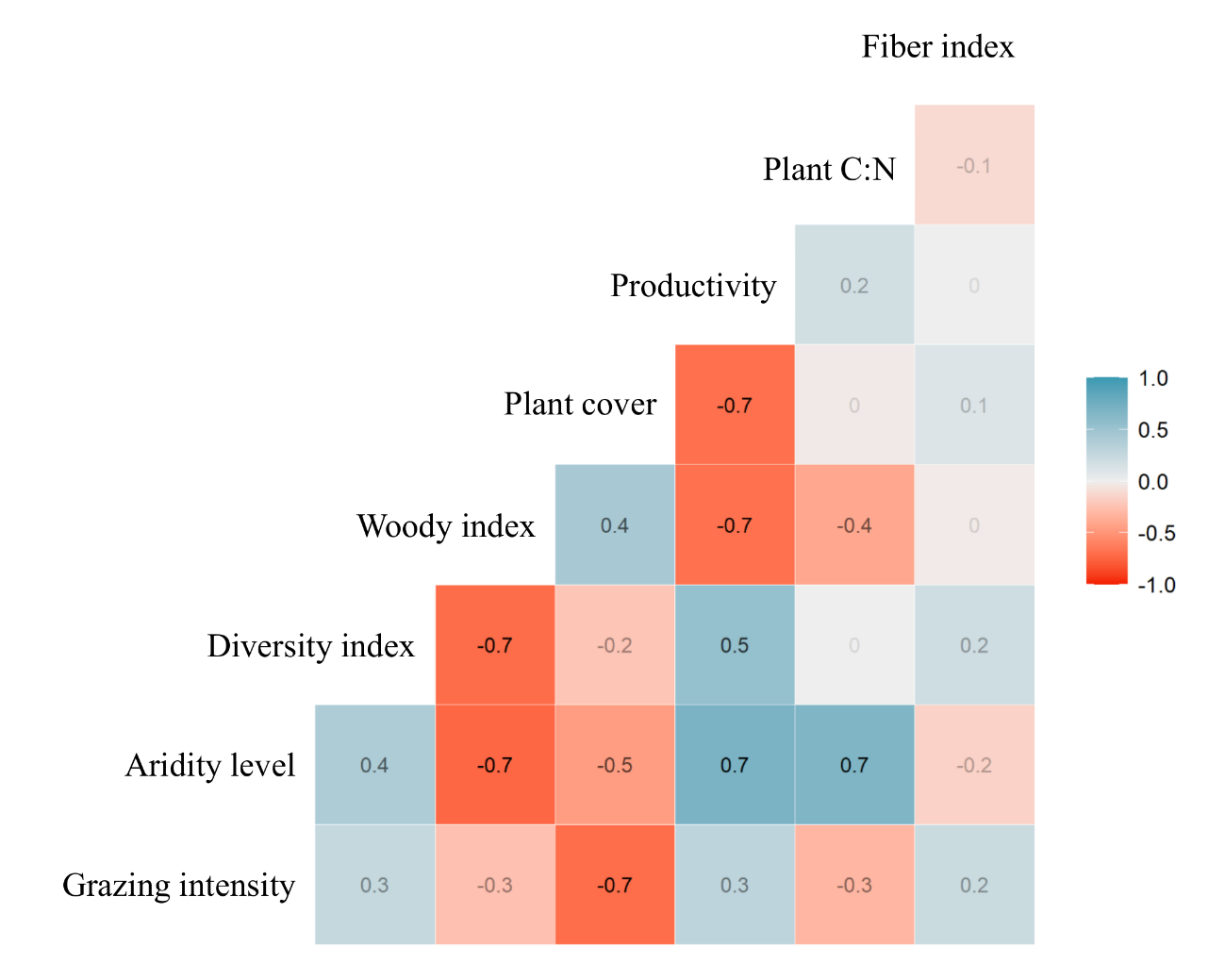


Fig. S5


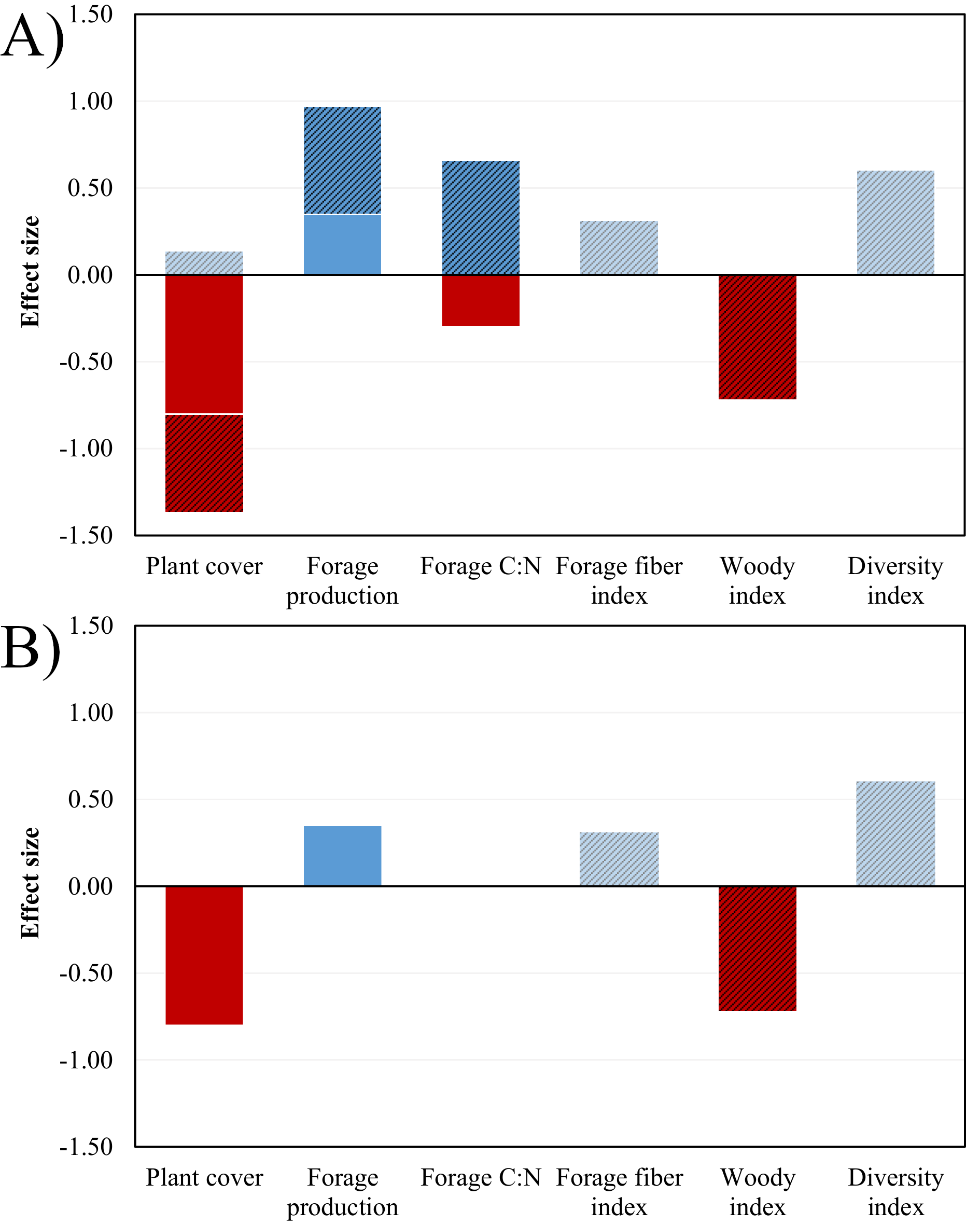


Table S2

|  |  |  | **Study plot** | | | | | | | | | | | | | | | | | |
| --- | --- | --- | --- | --- | --- | --- | --- | --- | --- | --- | --- | --- | --- | --- | --- | --- | --- | --- | --- | --- |
| **Plant species** | **Label** | **FV** | M1L | M1M | M1H | M2L | M2M | M2H | M3L | M3M | M3H | L1L | L1M | L1H | L2L | L2M | L2H | L3L | L3M | L3H |
| *Aegilops geniculata* | AEGI | 0-2 ^a,b^ | 0 | 0 | 0 | 0 | 0 | 0.1 | 0 | 0 | 0.2 | 0 | 0 | 0 | 0 | 0 | 0 | 0 | 0 | 0 |
| Annual 1 | ANU_1 | NA | 0 | 0 | 0 | 1.9 | 0 | 0 | 0 | 0 | 0 | 0 | 0 | 0 | 0 | 0 | 0 | 0 | 0 | 0 |
| Annual 2 | ANU_2 | NA | 0 | 0 | 0 | 0.6 | 0 | 0 | 0 | 0 | 0 | 0 | 0 | 0 | 0 | 0 | 0 | 0 | 0 | 0 |
| Annual 3 | ANU_3 | NA | 0 | 0 | 0 | 0.6 | 0 | 0 | 0 | 0 | 0 | 0 | 0 | 0 | 0 | 0 | 0 | 0 | 0 | 0 |
| Annual 4 | ANU_4 | NA | 0 | 0 | 0 | 0.1 | 0 | 0 | 0 | 0 | 0 | 0 | 0 | 0 | 0 | 0 | 0 | 0 | 0 | 0 |
| Annual 5 | ANU_5 | NA | 0 | 0 | 0 | 0.1 | 0 | 0 | 0 | 0 | 0 | 0 | 0 | 0 | 0 | 0 | 0 | 0 | 0 | 0 |
| Annual 6 | MEDIANU | NA | 0 | 0 | 0 | 0 | 0 | 0 | 0 | 0 | 0 | 0 | 0 | 0 | 0 | 0 | 0 | 0 | 0 | 0.1 |
| *Artemisia herba-alba* | ARTIBA | 2-4 ^a,b,c^ | 0 | 0.6 | 0 | 0 | 0 | 0 | 0 | 0 | 2.7 | 0 | 0 | 0 | 0 | 0 | 0.6 | 0 | 0 | 0 |
| *Asparagus horridus* | ASPHO | 0 ^a,b^ | 0 | 0 | 0 | 0 | 0 | 0.1 | 0 | 0 | 0 | 0 | 0 | 0 | 0 | 0 | 0 | 0 | 0 | 0 |
| *Asterolinon linum-stellatum* | ASTER | 1 ^b^ | 1.5 | 0 | 0 | 4.5 | 1.5 | 1.4 | 0.8 | 1.3 | 0 | 0.2 | 0 | 0 | 0 | 0 | 0 | 0.5 | 0.7 | 0.3 |
| *Astragalus stella* | ASTRA | 2-4 ^a,b^ | 0 | 0.7 | 0 | 0 | 0 | 0 | 0.2 | 0.1 | 0 | 0 | 0 | 0 | 0 | 0 | 0 | 0 | 0 | 0 |
| *Brachypodium retusum* | BRARE | 1 ^a,b,d^ | 0.1 | 4.9 | 1.4 | 25.5 | 7.5 | 0 | 0 | 4.2 | 2.9 | 0 | 6.2 | 0.6 | 0 | 0 | 3.3 | 0 | 0.1 | 0 |
| *Bromus rubens* | BRORU | 1-4 ^a,b,c^ | 0 | 0.8 | 0 | 2.8 | 0.8 | 0.7 | 0.1 | 0.9 | 0.8 | 0 | 0 | 0 | 0 | 0 | 0.1 | 0 | 0 | 0.6 |
| *Bupleurum semicompositum* | BUPLEU | 0-1 ^a,b^ | 0 | 1 | 0.1 | 0.1 | 0.1 | 0.3 | 0 | 0 | 0 | 0 | 0 | 0 | 0 | 0 | 0 | 0.1 | 0 | 0.2 |
| *Carlina corymbosa* | CACO | 0 ^b^ | 0 | 0.2 | 0 | 0 | 0 | 0 | 0 | 0.2 | 0.3 | 0 | 0 | 0 | 0 | 0 | 0.2 | 0 | 0 | 0.1 |
| *Catapodium rigidum* | DESMA | 0-2 ^a,b^ | 0.2 | 0.5 | 0.2 | 3 | 0.2 | 1.9 | 0.1 | 0.3 | 0.1 | 0.6 | 0 | 1.2 | 0 | 0 | 0.6 | 0.2 | 0 | 1.3 |
| *Chaenorrhinum rubrifolium* | CHAENO | NA | 0 | 0 | 0 | 0.3 | 0.1 | 0.1 | 0 | 0.1 | 0 | 0 | 0 | 0 | 0 | 0 | 0 | 0 | 0 | 0 |
| *Cistus clusii* | CISTUCLU | 0-2 ^a,b,e^ | 0 | 0 | 0 | 0 | 0 | 0 | 0 | 0 | 0 | 0 | 0 | 0 | 5.5 | 0 | 0 | 18 | 0 | 0.1 |
| Compositae 1 | COMPU_1 | NA | 0 | 0 | 0 | 0 | 0 | 0.1 | 0 | 0 | 0 | 0 | 0 | 0 | 0 | 0 | 0 | 0 | 0 | 0 |
| Compositae 2 | COMPU_2 | NA | 0 | 0 | 0 | 0.1 | 0 | 0.1 | 0 | 0 | 0 | 0 | 0 | 0 | 0 | 0 | 0 | 0 | 0 | 0 |
| Compositae 3 | COMPU_3 | NA | 0 | 0 | 0 | 0 | 0 | 0.1 | 0.1 | 0 | 0 | 0 | 0 | 0 | 0 | 0 | 0 | 0 | 0 | 0 |
| Compositae 4 | COMPU_4 | NA | 0 | 0 | 0 | 0.1 | 0 | 0 | 0 | 0 | 0 | 0 | 0 | 0 | 0 | 0 | 0 | 0 | 0 | 0 |
| Compositae 5 | COMPU_5 | NA | 0 | 0 | 0 | 0 | 0 | 0 | 0 | 0 | 0 | 0 | 0 | 0 | 0 | 0 | 0 | 0 | 0 | 0.2 |
| Compositae 6 | COMPU_6 | NA | 0 | 0 | 0 | 0 | 0 | 0 | 0 | 0 | 0 | 0 | 0 | 0 | 0 | 0 | 0 | 0 | 0 | 0.1 |
| Compositae 7 | COMPU_7 | NA | 0 | 0 | 0 | 0 | 0 | 0 | 0 | 0 | 0 | 0 | 0 | 0 | 0 | 0 | 0 | 0 | 0 | 0.1 |
| *Coris monspeliensis* | CORIS | 0 ^b,e^ | 0 | 0 | 0 | 0 | 0 | 0 | 0 | 0 | 0 | 0 | 0 | 0 | 0 | 0 | 0 | 0 | 0 | 0.1 |
| *Cuscuta sp.* | CUSCUTA | 0 ^a,e^ | 0 | 0 | 0 | 0 | 0 | 0 | 0 | 0 | 0 | 0 | 0 | 0 | 0 | 0.6 | 0 | 0 | 0.6 | 0 |
| *Dactylis glomerata L. subsp. hispanica* | DACT | 2 ^c^ | 1.3 | 0.2 | 0 | 2.2 | 0 | 0 | 0.6 | 0 | 0 | 0 | 0 | 0 | 0 | 0 | 0 | 0 | 0 | 0 |
| *Erodium cicutarium* | ERODIUN | 2 ^a^ | 0 | 0 | 0 | 0 | 0.1 | 0 | 0 | 0 | 0.1 | 0 | 0 | 0 | 0 | 0 | 0 | 0 | 0 | 0 |
| *Eruca vesicaria* | ERUCA | 1-2 ^a,b^ | 0 | 0 | 0 | 0 | 0 | 0 | 0 | 0.7 | 0 | 0 | 0 | 0 | 0 | 0 | 0 | 0 | 0 | 0 |
| *Euphorbia sp.* | EUPHORBIA | 0 ^a,b^ | 0 | 0 | 0 | 0.1 | 0 | 0 | 0 | 0 | 0 | 0 | 0 | 0 | 0 | 0 | 0 | 0 | 0 | 0 |
| *Filago pyramidata* | FILAGO | 0-1 ^a,b^ | 0.1 | 0.1 | 0 | 0.6 | 1.4 | 0.4 | 0.2 | 1.9 | 0 | 0 | 0 | 0.1 | 0 | 0 | 0.1 | 0.2 | 0 | 0.8 |
| *Fumana ericoides* | FUMERI | 0-2 ^b,f^ | 0 | 0 | 0 | 0 | 0 | 0 | 0 | 0 | 0 | 1.2 | 0 | 0.8 | 2.7 | 4.8 | 0.6 | 0 | 1.2 | 1.2 |
| *Galium verrucosum* | GALIUM | NA | 0.1 | 0.2 | 0 | 0.4 | 0.3 | 0.3 | 0.1 | 0.7 | 0 | 0.1 | 0 | 0 | 0 | 0 | 0.1 | 0.2 | 0.6 | 0.6 |
| *Genista scorpius* | GENISCO | 0-1 ^a,f^ | 0 | 0 | 0 | 8.2 | 1.8 | 0 | 0 | 0 | 0 | 4.9 | 2.7 | 0.6 | 2.1 | 12.9 | 2.8 | 2.1 | 3.4 | 0.2 |
| Grass 1 | GRAM_1 | NA | 0 | 0 | 0.1 | 0 | 0 | 0 | 0 | 0 | 0 | 0 | 0 | 0 | 0 | 0 | 0 | 0 | 0 | 0 |
| Grass 2 | GRAM_2 | NA | 0 | 0 | 0 | 0 | 0 | 0 | 0 | 0 | 0 | 0 | 0 | 0 | 0 | 0 | 0.1 | 0 | 0 | 0 |
| Grass 3 | GRAM_3 | NA | 0 | 0 | 0 | 0 | 0 | 0 | 0 | 0 | 0.1 | 0 | 0 | 0 | 0 | 0 | 0 | 0 | 0 | 0 |
| Grass 4 | GRAM_4 | NA | 0 | 0 | 0 | 2.4 | 0 | 0 | 0 | 0 | 0 | 0 | 0 | 0 | 0 | 0 | 0 | 0 | 0 | 0 |
| *Gypsophila struthium subsp. hispanica* | GYPSO | 0 ^g^ | 0 | 0 | 0 | 9.7 | 8.2 | 0.7 | 6.3 | 7.6 | 0.6 | 2.1 | 1.3 | 3.3 | 0 | 2.7 | 2.1 | 4.8 | 2.1 | 0 |
| *Hedypnois rhagadioloides* | HEDYP | 1 ^a,b^ | 0 | 0 | 0 | 0 | 0 | 0 | 0 | 0.1 | 0.1 | 0 | 0 | 0 | 0 | 0 | 0 | 0 | 0 | 0 |
| *Hedysarum humile* | HEDISA | 2 ^f^ | 0 | 0 | 0 | 0 | 0 | 0 | 1.2 | 0 | 0 | 0 | 0 | 0 | 0 | 0 | 0 | 0 | 0 | 0 |
| *Helianthemum hirtum* | HELIHIRT | 0-5 ^a,b,h^ | 0 | 0 | 0 | 0 | 0 | 0 | 0 | 0 | 0 | 0 | 0 | 0 | 0 | 0 | 0.2 | 0 | 0 | 0 |
| *Helianthemum ledifolium* | HELIPI | 0-4 ^a,b^ | 1.8 | 1.2 | 0 | 1.9 | 0 | 0 | 0 | 0 | 0 | 0 | 0 | 0 | 0 | 0 | 4 | 0 | 0 | 0.1 |
| *Helianthemum marifolium* | HELIMA | 0-3 ^b,f^ | 0.7 | 0 | 0 | 0.1 | 0.2 | 0.7 | 0 | 0 | 0.6 | 2 | 6.4 | 2.5 | 1.2 | 4.8 | 0 | 0.6 | 1.8 | 0.7 |
| *Helianthemum squamatum* | HELISQUA | 0 ^g^ | 0.7 | 3 | 2 | 2.4 | 1.9 | 0.5 | 19 | 2.5 | 0 | 0 | 0 | 0 | 0 | 0 | 0 | 0 | 0 | 0 |
| *Helianthemum syriacum* | HELILA | 0-4 ^b,g^ | 1.9 | 1.3 | 1.4 | 2.4 | 1.9 | 0.1 | 1.9 | 0 | 0 | 3.5 | 0 | 0 | 1.9 | 3.3 | 0.1 | 2.7 | 3 | 1 |
| *Helichrysum stoechas* | HELYCRI | 0-2 ^a,b,e^ | 0 | 0.1 | 1.5 | 1.8 | 1.4 | 0 | 1.8 | 0 | 0.1 | 2.3 | 3.5 | 0.7 | 0 | 0 | 0 | 0 | 1.9 | 1.2 |
| *Helictochloa gervaisii* | AVENA | NA | 0 | 0 | 0 | 0 | 0 | 0.6 | 0 | 0 | 0 | 0 | 0 | 0 | 0 | 0 | 0 | 0 | 0 | 0 |
| *Herniaria fruticosa* | HERNFRU | 0-1 ^b,g^ | 0.7 | 1.8 | 1.5 | 0.6 | 0.8 | 0.8 | 1.3 | 1.4 | 1.9 | 1.2 | 0.1 | 1.2 | 0.6 | 0.6 | 0 | 0 | 0.8 | 1.4 |
| *Hippocrepis ciliata* | HIPPO | 3-4 ^a,b^ | 0 | 0 | 0 | 0 | 0.1 | 0 | 0 | 0 | 0 | 0 | 0 | 0 | 0 | 0 | 0 | 0 | 0 | 0 |
| *Hordeum murinum* | HORDEUM | 1-3 ^a,b,c^ | 0 | 0 | 0 | 0 | 0 | 0 | 0 | 0 | 0 | 0 | 0 | 0 | 0 | 0 | 0 | 0 | 0 | 0.6 |
| *Koeleria vallesiana* | KOELERVAL | 1 ^a,b,f^ | 0.1 | 1.9 | 2.4 | 0.8 | 0.7 | 0.7 | 0.7 | 0.6 | 0.2 | 1.9 | 0.7 | 0.7 | 0 | 0.3 | 1.8 | 2.2 | 3.4 | 2.9 |
| *Launaea fragilis* | LAUNEA | 4-5 ^b^ | 0 | 0.1 | 0 | 0 | 0 | 0 | 0.1 | 0.7 | 0 | 0 | 0 | 0 | 0 | 0 | 0 | 0 | 0 | 0 |
| *Linum strictum* | LINU | 0-2 ^b,c^ | 0 | 0 | 0 | 0.6 | 0 | 0.1 | 0.3 | 0.4 | 0 | 0 | 0 | 0 | 0 | 0 | 0 | 0 | 0 | 0 |
| *Linum suffruticosum* | LINUSUB | 0-2 ^a,b,f^ | 0 | 0.2 | 0.6 | 0 | 0.1 | 0 | 0 | 0 | 0 | 6 | 1.8 | 2.5 | 0.2 | 0.7 | 0.1 | 0 | 1.4 | 3.4 |
| *Lygeum spartum* | LYGI | 1-2 ^a,b,c^ | 0 | 2.7 | 0 | 0 | 0.1 | 0 | 0 | 0 | 0 | 0 | 0 | 0 | 0 | 0 | 0 | 0 | 0 | 0 |
| *Neatostema apulum* | NEATOS | 0 ^b^ | 0 | 0.1 | 0 | 0.6 | 0.9 | 0.3 | 0 | 0.2 | 0 | 0 | 0.1 | 0 | 0 | 0 | 0 | 0 | 0 | 0 |
| *Ononis tridentata* | ONOTRI | 2 ^b,g^ | 0.6 | 0 | 0 | 2.1 | 7.6 | 0 | 0 | 0 | 0 | 14.5 | 4.1 | 0.2 | 15.2 | 0.1 | 0 | 2.1 | 4.1 | 7.8 |
| *Peganum harmala* | PEGANUM | 0-1 ^a,b,c^ | 0 | 0 | 0 | 0.6 | 0.1 | 0 | 0 | 0 | 0 | 0 | 0 | 0 | 0 | 0 | 0 | 0.6 | 0.2 | 1.4 |
| *Plantago afra* | PLAFRA | 0-3 ^a,b,f^ | 0 | 0.2 | 0 | 0 | 0 | 0 | 0 | 0 | 0 | 0 | 0 | 0 | 0 | 0 | 0 | 0 | 0 | 0 |
| *Plantago albicans* | PLAL | 1-5 ^a,b,f^ | 0 | 1.5 | 0.7 | 0 | 0.2 | 0.4 | 2.5 | 3.5 | 4 | 0 | 0 | 0 | 0 | 0 | 0 | 0 | 0 | 0 |
| *Polygala rupestris* | POLYGALA | 3 ^b^ | 0.7 | 0 | 0 | 0 | 0 | 0 | 0 | 0 | 0 | 0.4 | 1.2 | 0 | 1.5 | 0.1 | 0 | 1.9 | 0.2 | 0 |
| *Rosmarinus officinalis* | ROME | 0-3 ^a,b,f^ | 25.5 | 0 | 0 | 0 | 0 | 0 | 0 | 0 | 0 | 36 | 31 | 26.9 | 48 | 25.5 | 18.5 | 27 | 6.2 | 4.3 |
| *Salsola vermiculata* | SAVE | 2-4 ^a,b,c^ | 0 | 2.1 | 0 | 0 | 0 | 0 | 0 | 0 | 0 | 0 | 0 | 0 | 0 | 0 | 0 | 0 | 0 | 0 |
| *Salvia lavandulifolia* | SALVIA | 0-1 ^a,e^ | 0 | 0 | 0 | 0 | 0 | 0 | 0 | 0 | 0 | 0 | 0 | 0 | 0 | 0 | 0 | 0 | 0.7 | 0 |
| *Scorzonera aristata* | SCORZO | NA | 0 | 0 | 0.8 | 0 | 0.2 | 0.1 | 0 | 0 | 0.1 | 0.7 | 0 | 0 | 0 | 0 | 0 | 0 | 0 | 0 |
| *Sedum sediforme* | SEDUM | 0-1 ^a,b^ | 0 | 0.1 | 0 | 0.1 | 0 | 0 | 0 | 0 | 0 | 0 | 0 | 0 | 0 | 0 | 0 | 0 | 0 | 0 |
| *Sideritis hirsuta* | SIDEHIR | 0 ^e^ | 0 | 1.3 | 0.8 | 0.8 | 2.2 | 0.1 | 0 | 2.2 | 0.7 | 0 | 0 | 0 | 0 | 0 | 0 | 0 | 0 | 0 |
| *Stipa lagascae* | STIPALAG | 1-4 ^a,b,c^ | 0.6 | 0.8 | 0 | 1.8 | 3.9 | 4 | 12.4 | 8.9 | 0.8 | 0.1 | 0.1 | 0.7 | 6.9 | 0 | 0.6 | 0 | 12.5 | 2.7 |
| *Teucrium capitatum* | TEUCAP | 0-2 ^a,b,g^ | 0.2 | 0 | 1.3 | 0 | 0 | 0 | 0.1 | 0 | 0.6 | 3 | 0 | 0.1 | 0 | 0 | 1.2 | 0.6 | 1.8 | 4 |
| *Thymelaea nivalis* | THYMELEA | 0 ^g^ | 0 | 0 | 0 | 0 | 0 | 0 | 0 | 0 | 0 | 2.1 | 0 | 0 | 0 | 0 | 0 | 0 | 0 | 0.6 |
| *Thymus vulgaris* | THYMUVU | 0-1 ^a,b,g^ | 0.3 | 1.8 | 2.3 | 9 | 8 | 5 | 4 | 0.8 | 0 | 6 | 3.4 | 4.4 | 0.6 | 16 | 1.2 | 3.3 | 2.8 | 0.3 |
| *Trachynia distachya* | BRADI | 0-4 ^a,b,c^ | 0 | 0 | 0 | 0 | 0 | 0 | 0 | 0 | 0.1 | 0 | 0 | 0 | 0 | 0 | 0 | 0 | 0 | 0 |
| Unknown sp. | HEDYSA_2 | NA | 0 | 0 | 0 | 0 | 0 | 0 | 0.6 | 0 | 0 | 0 | 0 | 0 | 0 | 0 | 0 | 0 | 0 | 0 |
| **Total richness** |  |  | 18 | 27 | 15 | 35 | 28 | 26 | 22 | 22 | 20 | 20 | 14 | 16 | 12 | 13 | 20 | 17 | 21 | 30 |

^a^ Daget Philippe, Poissonet Jacques, Huguenin Johann. 2010. Prairies et Pâturages - Méthodes d'étude de terrain et interprétations. Montpellier: CIRAD, 955 p. https://umr-selmet.cirad.fr/publications-et-ressources/documents-techniques

^b^ Roggero, P.P.; Bagella, S.; Farina, R. 2002. Un archivio dati di Indici specifici per la valutazione integrata del valore pastorale. Riv. Agron., 36, 149–156.

^c^ Barrantes, O., Reiné, R., Ascaso, J., Mendoza, A., Broca, A., Ferrer, C., 2004. Pastizales (albardinales) y pastos arbustivos xerófilos (sisallares) de la depresión del Ebro en la provincia de Huesca. Tipificación, cartografía y valoración. In: Pastos y ganadería extensiva. (Ed. García-Criado B., García-Ciudad A., Vázquez de Aldana B. R., Zalbagogeazcoa I.), 607-612. Ed. IRNA-CSIC, Salamanca (España).

^d^ Barrantes, O.; Reiné, R.; Broca, A.; Gonzalo, S.; Ascaso, J.; Ferrer, C., 2005. Pastos arbustivos de coscojar y de espinar caducifolio en la Cordillera Ibérica de Aragón. Tipificación, cartografía y valoración. In: Producciones agroganaderas: gestión eficiente del medio natural. De la Roza B., Martínez A., Carballal A. (Eds.). Ed. SERIDA, Asturias. Pp. 747-754. ISBN: 84-611-2820-6

^e^ Reiné, R., Barrantes, O., Ascaso J., Mendoza, A., Broca, A., Ferrer, C., 2004. Pastos arbustivos (erizonales, romerales-aliagares-lastonares y matorrales espinosos con boj) del prepirineo en la provincia de Huesca. Tipificación, cartografía y valoración. En: Pastos y ganadería extensiva. (Ed. García-Criado B., García-Ciudad A., Vázquez de Aldana B. R., Zalbagogeazcoa I.), 625-630. Ed. IRNA-CSIC, Salamanca (España).

^f^ Barrantes, O., Reiné, R., Ascaso, J., Mendoza, A., Broca, A., Ferrer, C., 2004. Pastos arbustivos y pastizales del tipo lasto-timo-aliagar de la depresión del Ebro en la provincia de Huesca. Tipificación, cartografía y valoración. In: Pastos y ganadería extensiva. (Ed. García-Criado B., García-Ciudad A., Vázquez de Aldana B. R., Zalbagogeazcoa I.), 601-606. Ed. IRNA-CSIC, Salamanca (España).

^g^ Barrantes, O., Reiné, R., Ascaso, J., Mendoza, A., Broca, A., Ferrer, C., 2004. Pastos arbustivos gipsófilos y halófilos de la depresión del Ebro en la provincia de Huesca. Tipificación, cartografía y valoración. In: Pastos y ganadería extensiva. (Ed. García-Criado B., García-Ciudad A., Vázquez de Aldana B. R., Zalbagogeazcoa I.), 613-618. Ed. IRNA-CSIC, Salamanca (España).

^h^ Reiné, R.; Barrantes, O.; Broca, A.; Gonzalo, S.; Ascaso, J.; Ferrer, C., 2005. Pastos arbustivos de erizal y de jaral en la Cordillera Ibérica de Aragón. Tipificación, cartografía y valoración. In: Producciones agroganaderas: gestión eficiente del medio natural. De la Roza B., Martínez A., Carballal A. (Eds.). Ed. SERIDA, Asturias. Pp. 763-770. ISBN: 84-611-2820-6
